# Comprehensive Analysis Reveals the Difference in Volatile Oil between *Bupleurum marginatum* var. *stenophyllum* (Wolff) Shan et Y. Li and the Other Four Medicinal *Bupleurum* Species

**DOI:** 10.3390/molecules29112561

**Published:** 2024-05-29

**Authors:** Yuzhi Ma, Xinwei Guo, Peiling Wu, Yuting Li, Ruyue Zhang, Lijia Xu, Jianhe Wei

**Affiliations:** 1 Key Laboratory of Bioactive Substances and Resources Utilization of Chinese Herbal Medicine, Ministry of Education and National Engineering Laboratory for Breeding of Endangered Medicinal Materials, Institute of Medicinal Plant Development, Chinese Academy of Medical Sciences and Peking Union Medical College, Beijing 100193, China; mayuzhi@implad.ac.cn (Y.M.); xwguo@implad.ac.cn (X.G.); wupeiling@implad.ac.cn (P.W.); liyuting@implad.ac.cn (Y.L.); zhangruyue@implad.ac.cn (R.Z.); 2 Hainan Provincial Key Laboratory of Resources Conservation and Development of Southern Medicine, Hainan Branch of the Institute of Medicinal Plant Development, Chinese Academy of Medical Sciences and Peking Union Medical College, Haikou 570311, China

**Keywords:** *Bupleurum*, volatile oil, GC-MS, phytochemistry, e-nose

## Abstract

Volatile oil serves as a traditional antipyretic component of Bupleuri Radix. *Bupleurum marginatum* var. *stenophyllum* (Wolff) Shan et Y. Li belongs to the genus *Bupleurum* and is distinguished for its high level of saikosaponins and volatile oils; nonetheless, prevailing evidence remains inconclusive regarding its viability as an alternative resource of other official species. This study aims to systematically compare the volatile oil components of both dried and fresh roots of *B. marginatum* var. *stenophyllum* and the four legally available *Bupleurum* species across their chemical, molecular, bionics, and anatomical structures. A total of 962 compounds were determined via GC-MS from the dried roots; *B. marginatum* var. *stenophyllum* showed the greatest differences from other species in terms of hydrocarbons, esters, and ketones, which was consistent with the results of fresh roots and the e-nose analysis. A large number of DEGs were identified from the key enzyme family of the monoterpene synthesis pathway in *B. marginatum* var. *stenophyllum* via transcriptome analysis. The microscopic observation results, using different staining methods, further showed the distinctive high proportion of phloem in *B. marginatum* var. *stenophyllum*, the structure which produces volatile oils. Together, these pieces of evidence hold substantial significance in guiding the judicious development and utilization of *Bupleurum* genus resources.

## 1. Introduction

*Bupleurum* L. comprises approximately 248 accepted species globally, with a total of 25 species, 8 varieties, and 3 variants identified in China, the majority of which have been used as traditional medicines for more than 2000 years [1]. Despite a lot of *Bupleurum* species being documented for medicinal use, currently, only four species are designated for legal use in China, namely, *Bupleurum chinense* DC., *Bupleurum scorzonerifolium* Willd., *Bupleurum smithii* Wolff, and *Bupleurum marginatum* Wall. ex DC. These four species constitute the primary medicinal constituents of Bupleuri Radix in the market [2]. *B. chinense* DC. and *B. scorzonerifolium* Willd. are the sole species officially recognized by the *Chinese Pharmacopoeia*; while *B. smithii* Wolff and *B. marginatum* Wall. ex DC. are listed in the local standards of several Provinces of China. Unlike traditional crops that have been domesticated by humans for thousands of years, the use of medicinal plants has long relied on wild resources, with a short history of simple artificial cultivation and very few germplasms with clear genetic backgrounds [3]. Multiple studies have shown that the environment is still the primary factor affecting the quality of medicinal materials; therefore, genuine producing areas are regarded as the most important source of high-quality medicinal materials [4]. It has been shown that the genuine producing area of *B. chinense* DC. is the Shaanxi Province of China [5,6]. *B. scorzonerifolium* Willd. was once widely distributed in the Central, Southern, and Northern China, but after changes in—and the contraction of—the producing areas, it is currently mainly concentrated in Heilongjiang Province of China [7,8]. The genuine producing area and the local standards inclusion area of *B. smithii* Wolff is Northwest China, and currently, Shanxi Province is one of its best and largest producing areas [1]. *B. marginatum* Wall. Ex DC. is mainly distributed in the southwest and central parts of China; its genuine producing areas and the local standards inclusion areas mainly include the Sichuan, Guizhou, and Yunnan Provinces of China [9,10].

*B. marginatum* var. *stenophyllum* (Wolff) Shan et Y. Li is a variety of *B. marginatum* Wall. ex DC. Although it is illegal to use it as Bupleuri Radix, *B. marginatum* var. *stenophyllum* has been widely cultivated in Gansu Province, China, because of its extremely high level of saikosaponins as compared with other *Bupleurum* species/varieties [11,12]. Some studies have shown that the main chemical components of *B. marginatum* var. *stenophyllum* include saikosaponins, volatile oils, flavonoids, etc. In a recent study, there was no significant difference observed in the levels of the predominant flavonoids such as quercetin, isorhamnetin, and kaempferide between *B. marginatum* var. *stenophyllum* and *B. chinense* DC. or *B. scorzonerifolium* Willd [13]. However, the research on the volatile oil of *B. marginatum* var. *stenophyllum* is still limited, with only a few volatile components identified in its seeds through GC-MS, including D (+)-carvone, dipentene, oxidized caryophyllene, undecane, 3-isopropenyl-5,5-dimethyl-cyclopentene, heptaldehyde, and 3-carene [14].

The *Chinese Pharmacopoeia* stipulates that Bupleuri Radix can be identified via thin-layer chromatography with saikosaponin A and D as indicators. However, almost all *Bupleurum* contains saikosaponin A and D, especially *B. marginatum* var. *stenophyllum*. Because of its high content of saikosaponins, some merchants have substituted the two *Bupleurum* species in the *Chinese Pharmacopoeia* with *B. marginatum* var. *stenophyllum* in the market. Nonetheless, owing to the limited research on the chemical composition and pharmacological effects of *B. marginatum* var. *stenophyllum*, there remains insufficient evidence to determine whether *B. marginatum* var. *stenophyllum* is similar in composition and efficacy to other legitimate *Bupleurum* species and if it can be regarded as an alternative resource of the two species which were officially recognized by the *Chinese Pharmacopoeia.*

It is well known that the volatile oil of certain aromatic medicinal plants, including *Ligusticum sinense “Chuanxiong”* and *Chrysanthemum* × *morifolium* (Ramat.) Hemsl, possess an antipyretic effect [15,16]. The *Bupleurum* species, originating from the *Apiaceae* family, also belongs to the domain aromatic plants. Historically, its volatile oil has been recognized as the traditional antipyretic component of Bupleuri Radix [17]. To date, approximately 160 known volatile oil components with identified structures have been reported in the genus *Bupleurum*, including lauraldehyde, *β*-Pinene, hexanal, and so on [18]. As early as the 1940s, the volatile oil of Bupleuri Radix was successfully extracted, and its anti-influenza virus properties were confirmed through in vitro experiments, making it the earliest TCM injection listed in China [19]. Contemporary pharmacological studies have further elucidated the effects of volatile oil, such as its antipyretic, analgesic, anti-inflammatory, and anticonvulsant effects [1]. Due to its low molecular weight and instability, the extraction process of the volatile oil from *Bupleurum* species/varieties poses considerable challenges. Current studies on the volatile oil are predominantly focused on *B. chinense* DC. and *B. scorzonerifolium* Willd., and there is still a lack of systematic study on the volatile oil components across the principal medicinal species of the genus *Bupleurum* [20]. Specifically, a comparative analysis regarding the volatile oil composition between *B. marginatum* var. *stenophyllum* and all legally available *Bupleurum* species remains absent.

In light of this, the present study aims to systematically compare the differences in the volatile oil components between *B. marginatum* var. *stenophyllum* and the four legally available *Bupleurum* species previously mentioned. The comparison will be conducted on both dried and fresh roots, utilizing techniques such as GC-MS, RNA seq, electronic nose analysis, and frozen section. The relevant results will provide an important basis for objectively understanding and utilizing the medicinal value of five *Bupleurum* species/varieties, especially *B. marginatum* var. *stenophyllum*. This, in turn, will help in considering the further utilization of the five *Bupleurum* species/varieties.

## 2. Results

### 2.1. DNA Barcoding Analysis

In this study, a total of seven plant materials which belong to four main medicinal species of the genus *Bupleurum* were included (Figure S1). We first performed DNA barcoding analysis with the previously reported primer ITS to identify whether the species/varieties of the plant material used are correct [5]. As shown in Figure S1, the origins of all the plant materials were proved to be correct. Consistent with previous results on the taxonomy of the genus *Bupleurum*, *B. marginatum* Wall. ex DC. and *B. marginatum* var. *stenophyllum* were grouped into one cluster, and *B. chinense* DC., *B. scorzonerifolium* Willd., and *B. smithii* Wolff were grouped separately.

### 2.2. E-Nose Analysis of the Dried Roots

**Figure 1:**
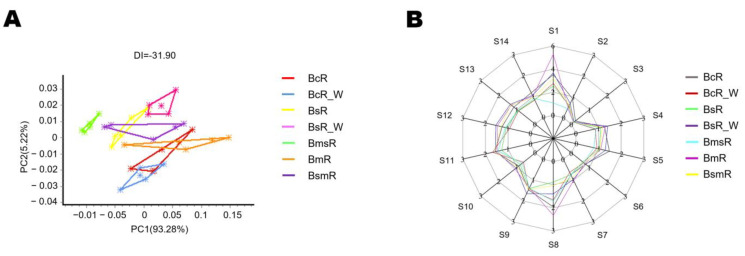
The e-nose analysis of the dried samples of the five species/varieties of Bupleuri Radix: (**A**) the PCA analysis based on the original data of e-nose detection; (**B**) the response of each sample to different sensors. (S1, ammonia, amines; S2, hydrogen sulfide, sulfide; S3, hydrogen; S4, ethanol; S5, alcohols, ketones, aldehydes, aromatic compounds; S6, methane, biogas, natural gas; S7, combustible gas; S8, volatile organic compounds (VOCs); S9, liquefied gas, natural gas, gas; S10, liquefied gas, combustible gas; S11, alkanes, ethanol, natural gas, smoke; S12, organic solvents; S13, smoke, cooking odor; S14, methane, gas.)

The PCA analysis based on the original data of e-nose detection (Table S2) is shown in Figure 1A. Among the five species/varieties of Bupleuri Radix, the dried samples of *B. marginatum* var. *stenophyllum* were clearly distinguished from all other samples; the dried samples of *B. marginatum* Wall. ex DC. and *B. chinense* DC., as well as *B. smithii* Wolff and *B. scorzonerifolium* Willd., were more similar in terms of odor, which were all consistent with the PCA results of the metabolome analysis based on dried samples.

The response of each sample to different sensors is shown in Figure 1B. The responses of *B. marginatum* var. *stenophyllum* on sensor 1 and sensor 5, which represents ammonia and amines, and alcohols, ketones, aldehydes, and aromatic compounds, respectively, were significantly lower than those of all other samples. This was consistent with the results of metabolomics analysis based on dried samples, where *B. marginatum* var. *stenophyllum* showed the greatest difference in aromatic compounds such as alcohols, aldehydes, ketones, and esters compared to other species. Since ammonia water has the disinfection effect, the difference in the ammonia response might be related to the fact that *B. marginatum* var. *stenophyllum* might not have undergone strict disinfection and sterilization due to its illegal marketing qualification. Overall, all the samples showed the most significant differences in response to sensor 1, sensor 5, and sensor 8, which all represents volatile compounds, indicating that volatile oil does indeed constitute an important difference among the different *Bupleurum* species/varieties.

### 2.3. Metabolomics Profiling of Different Dried Roots

**Figure 2:**
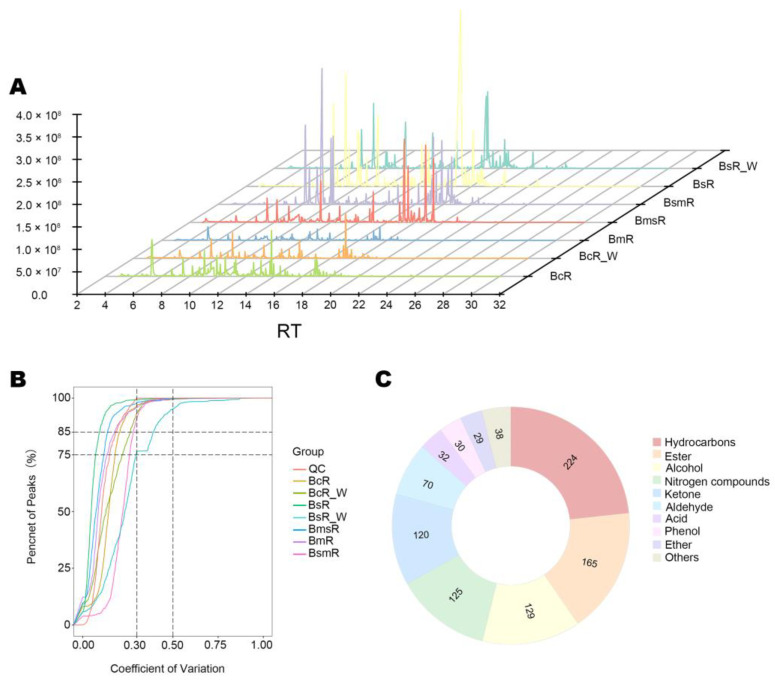
Total ion chromatogram (TIC) of (**A**) dried samples and quality control (QC) sample with the coefficient of variation (CV) of (**B**) dried samples. (**C**) Doughnut chart of the number of different volatile metabolite types of dried samples.

HS-SPME combined with GC-MS was used to explore the differences in volatile metabolite composition between five *Bupleurum* species/varieties. The total ion chromatogram is shown in (Figure 2A). The coefficient of variation (CV) of 75% peaks is less than 0.3 in the QC sample, which shows the data were stable. (Figure 2B). A total of 962 compounds were detected in dried roots, including 224 hydrocarbons, 165 esters, 129 alcohols, 125 nitrogen compounds, 120 ketones, 70 aldehydes, 32 acids, 30 phenols, 29 ethers, and 38 other compounds (Figure 2C). Details of the metabolites are shown in Table S3.

**Figure 3:**
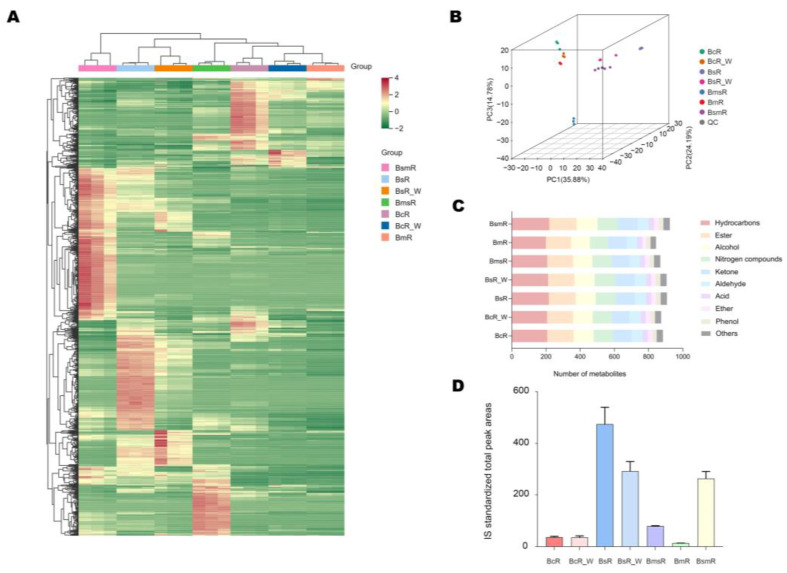
Overview of the detected metabolites in the five species/varieties: (**A**) heat map of HCA of dried samples. In the heatmap, color-coding consists of shades of red and green, in which a higher intensity of red stands for very high concentration, and a higher intensity of green stands for very low concentration; (**B**) the 3D PCA plot of dried samples; (**C**) distribution of different types of metabolites in dried samples; (**D**) IS standardized total peak areas of volatile metabolites in different samples.

We used a variety of statistical methods to assess the differences between the five *Bupleurum* species/varieties. HCA showed the different relationship in chemical composition between five *Bupleurum* species/varieties. In the heatmap, there was a closer relationship between BcR, BcR_W, and BmR. BmsR was grouped with BcR, BcR_W, and BmR. BsR, BsR_W, and BsmR are far away from BcR, BcR_W, BmR, and BmsR (Figure 3A).

In the 3D PCA plot, the different samples were separated, with PC1, PC2, and PC3, accounting for 35.88%, 24.19%, and 14.78%, respectively (Figure 3B). The results showed that there was an obvious separation trend among different samples, and the BmsR was separated from the other samples on PC3, accounting for 14.78% of the total variance.

There were 884, 874, 908, 906, 869, 844, and 925 volatile metabolites detected in the dried samples of BcR, BcR_W, BsR, BsR_W, BmsR, BmR, and BsmR, respectively. Approximately 692 metabolites were commonly detected in all five species/varieties. The metabolite proportions of all samples were similar. Hydrocarbons, esters, and alcohols were the major metabolite classes (Figure 3C). We represent the content of the samples via the IS standardized total peak areas. The contents of volatile metabolites in different samples were different, and BsR and its wild sample were the highest, followed by BsmR and BmsR (Figure 3D).

### 2.4. Identification of Metabolites Responsible for Differences

The difference between BmsR and the other four species of *Bupleurum* mainly lies in some individual components and their contents. To find the different compounds between the five *Bupleurum* species/varieties, the supervised method, OPLS-DA, and Student’s *t*-test were used.

**Figure 4:**
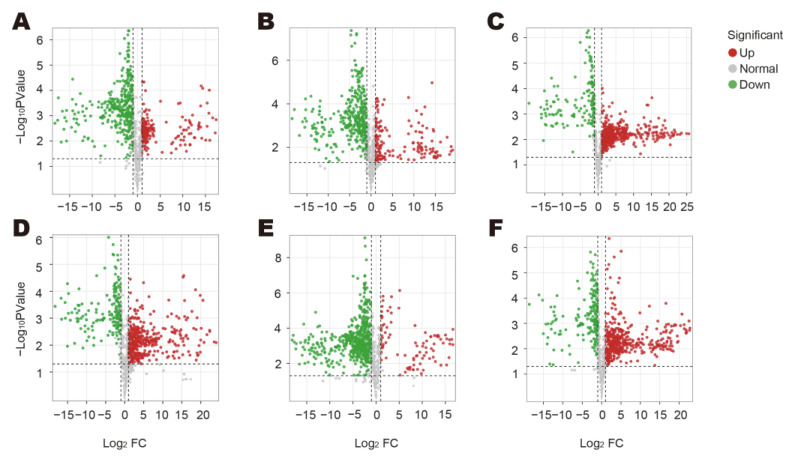
Volcano plots of differential metabolites in dried samples: (**A**) BcR vs. BmsR; (**B**) BcR_W vs. BmsR; (**C**) BsR vs. BmsR; (**D**) BsR_W vs. BmsR; (**E**) BmR vs. BmsR; (**F**) BsmR vs. BmsR.

To clarify the discrepancy in the volatile metabolites among the five *Bupleurum* species/varieties, the key differences between BmsR and the other four species/varieties were explored, respectively (FC ≥ 2 or FC ≤ 0.5, value of *p* < 0.05 and VIP ≥ 1) (Figure 4A–F and Table S4).

A comparative analysis was conducted between BmsR and the other species. The contents of some metabolites amount to the main differences among the five species/varieties. In BcR, the relative contents of hexanal, *o*-cymene, (2*E*,4*E*)-nona-2,4-dienal, and 2-isopropyl-1-methoxy-4-methylbenzene were higher and were significantly up-regulated compared with BmsR. The relative contents of 2,4-hexadienal, (2*E*,4*E*)-nona-2,4-dienal, dodecanal, 4-pentenyl acetate, *n*-decyl acetate, and other compounds were higher in the BcR_W and were significantly up-regulated compared with BmsR. Compared to BmsR, the relative contents of sabinen, *β*-pinene, *n*-decyl acetate, *α*-pinene, dodecanal, *o*-cymene, *β*-phellandrene, citral, and *γ*-terpinene were higher in BsR and its wild samples and significantly up-regulated. In BmR, the relative contents of 2-methyltetrahydrothiophen-3-one, heptanal, *β*-pinene, sabinene, and benzyl angelate were higher, and *α*-maaliene, *β*-elemen, and dodecanal were significantly up-regulated compared to those of BmsR. Compounds with higher relative content in BsmR, such as *α*-pinene, sabinen, *β*-pinene, *β*-myrcene, *β*-guaiene, 2-amylfuran, and so on, were almost all significantly up-regulated when compared to BmsR.

Moreover, we also identified 66 metabolites that were significantly up-regulated in BmsR compared with other species, mainly including esters, hydrocarbons, and ketones; the top 20 characteristic differential metabolites (arranged by the standardized peak area) are shown in Table 1.

### 2.5. Transcriptome Sequencing, Assembly, and Annotation of the Fresh Samples

We also identified the metabolites in fresh roots (Figure S2 and Table S5). There were 852, 756, 751, 835, 789, 762, and 822 metabolites detected in the fresh roots of BcR, BcR_W, BsR, BsR_W, BmsR, BmR, and BsmR, respectively. In the fresh samples, PC1, PC2, and PC3 accounted for 32.66%, 20.06%, and 16%, respectively. We also screened out different metabolites between BmsR and other species. Compared with the other four species/varieties, the contents of hydrocarbons, ketones, and esters in BmsR were relatively up-regulated (Figure S3 and Table S6).

To investigate the expression differences of the key genes involved in the biosynthesis of the related components of volatile oil in different *Bupleurum* species/varieties, 21 RNA-Seq libraries (5 different species/varieties, 3 biological repeats) were constructed. After removing the repeats with the low correlation values (<0.6) with other repeats, 16 RNA-Seq libraries were finally obtained (three repeats for *B. scorzonerifolium* Willd. (wild) and *B. marginatum* var. *stenophyllum*, respectively; two repeats for other species/varieties). After sequencing and filtering the raw reads, 19.05~25.49 million clean reads were obtained, with the GC contents ranging from 42.55% to 43.27%, respectively (Table S7). The Q30 value of each sample was all higher than 92.09%. A total of 99,960 unigenes were assembled with the N50 value of 2105 bp (Table S8). Finally, 21,863, 56,228, 46,059, 38,866, 52,190, 45,450, 65,470, 52,819, and 71,495 assembled unigenes were annotated by the GO, COG, KEGG, Pfam, Swissprot, KOG, eggnog, TrEMBL, and Nr databases, respectively (Table S9). The RNA-Seq data were available on NCBI (accession number PRJNA981284).

**Figure 5:**
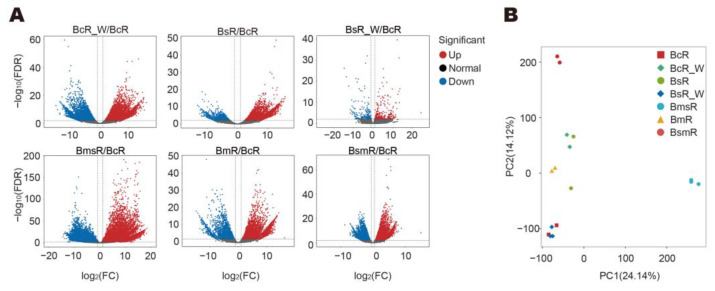
The bioinformatic analysis of the transcriptome data of the fresh samples of the five species/varieties of the genus *Bupleurum*. (**A**) The volcano plot of the differentially expressed genes in each fresh sample of the 6 species/varieties. Fresh samples of *B. chinense* was used as control. (**B**) The PCA analysis is based on transcriptome data of the five species/varieties.

In line with the metabolomic results of fresh samples, when using BcR as control, BmsR showed the highest number of DEGs at 28,996, while BsR_W was the closest to BcR, with only 533 DEGs. The number of DEGs between BsR, BcR_W, and BcR was 4625 and 10,613, respectively, suggesting the relatively high similarity between BcR and BsR (Figure 5A and Table S10). Accordingly, as shown in the PCA results, BmsR was significantly separated from all the samples; *B. chinense* DC. and *B. scorzonerifolium* Willd. exhibited cross similarity between their wild and cultivated samples (Figure 5B).

### 2.6. DEGs Involved in the Synthesis or Metabolism of Volatile Substances in B. marginatum var. stenophyllum

Next, we conducted transcriptome sequencing to explore the possible molecular mechanism that contributes to the different levels of related compounds among the seven samples.

Generally, the biosynthetic pathways of volatile compounds can be divided into three pathways: terpenoid pathway, amino acid pathway, and fatty acid pathway. We found that many differential metabolites belong to terpenoids. Based on the metabolomics results, we focused on the genes involved in the biosynthesis or metabolism of terpenoids, ketones, and esters.

**Figure 6:**
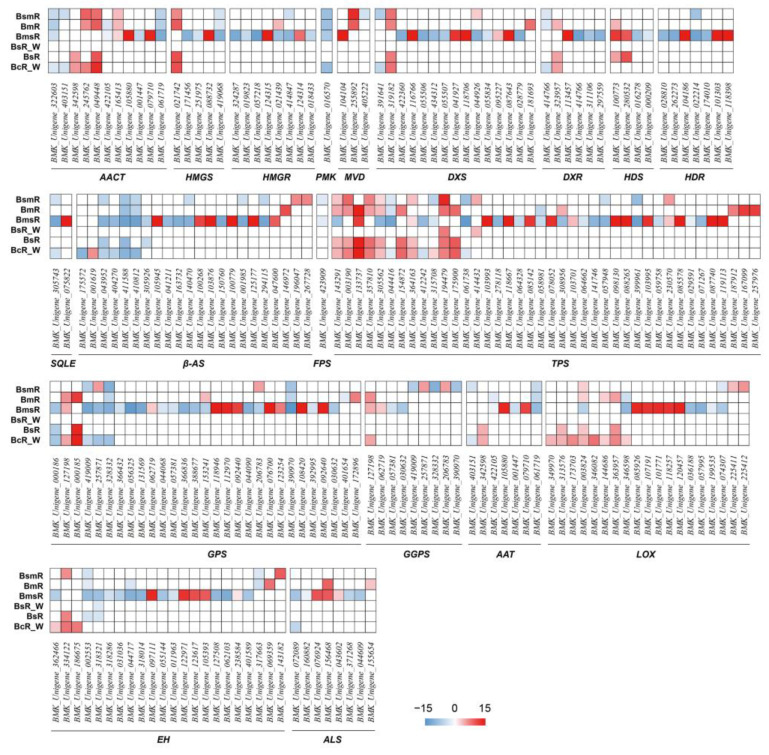
The differentially expressed genes among the fresh samples of the five species/varieties of the genus *Bupleurum*. Fresh samples of *B. chinense* were used as control.

Numerous key enzyme genes of triterpene saponin biosynthesis have been reported in *B. chinense*; many of key enzyme genes, such as TPS, AACT, DXS, GPS, GGPS, etc., exhibited significant differential expressions in this study (Figure 6). Only TPSs, which have pivotal roles in the biosynthesis of triterpenes and monoterpenes, showed relatively consistent up-regulation expression in all samples; the differentially expressed TPSs in *B. scorzonerifolium* Willd were all up-regulated. However, another large group of TPSs were only differentially expressed in *B. marginatum* var. *stenophyllum* and exhibited down-regulation. Similar situations were also found in several other families such as HDS or AACT. The above results were consistent with the high level of monoterpenes such as pinene in *B. scorzonerifolium* Willd, suggesting that TPS is a key gene for the synthesis of monoterpenes in volatile oil of the genus *Bupleurum*.

The key enzymes in the fatty acid and amino acid pathways are mainly lipoxygenase (LOX), alcohol dehydrogenase (ADH), hydrogen peroxide lyase (HPL), and alcohol acyltransferase (AAT). In the fatty acid pathway, substances such as aldehydes, enals, and alcohols are synthesized through the LOX/HPL pathway, followed by the formation of esters. ADH is one of the key enzymes that promote the mutual conversion between alcohols and aldehydes or ketones. Alcohol acyltransferase (AAT) is a key enzyme in the amino acid pathway that promotes the formation of branched chain esters. Easters are a type of enzyme that catalyzes ester hydrolysis reactions. Similar to the situation with TPSs, a large number of unique DEGs were identified in *B. marginatum* var. *stenophyllum*, and their overall expression patterns are opposite to other species. For example, LOX, ADH, and AAT were mostly up-regulated in *B. marginatum* var. *stenophyllum* but down-regulated in other species, while EH was the opposite. No differential expression of HPL was found in all samples.

In terms of the expression pattern and expression changes of the above DEGs, the difference between BsR, BsR_W, and BcR was the smallest, followed by BcR_W and BcR, indicating the little difference in fresh samples between *B. chinense* DC. and *B. scorzonerifolium* Willd.; the difference between BmR and BcR_W—that is, *B. marginatum* Wall. ex DC. and *B. chinense* DC.—was also small. All results were consistent with the PCA analysis of both transcriptome and metabolome sequencing based on fresh samples but differ from the metabolome PCA results based on dried samples; for example, the significant difference between *B. chinense* DC. and *B. scorzonerifolium* Willd. Part of the above results have been verified via qRT-PCR (Figure S4).

### 2.7. Microstructure of the Root Cross-Section of the Fresh Samples

The calculation of the area ratio of xylem to phloem showed that *B. chinense* DC. and *B. marginatum* Wall. Ex DC. Had the highest ratios of 0.3991 and 0.3643, respectively; *B. scorzonerifolium* Willd. Was 0.3290, *B. smithii* Wolff was 0.2578, while *B. marginatum* var. *stenophyllum* had the lowest ratio of 0.2253.

**Figure 7:**
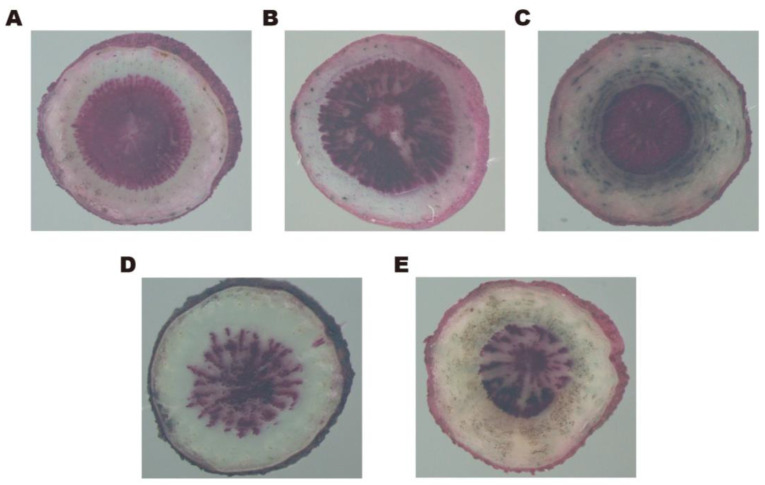
The samples were stained by hydrochloric acid phloroglucinol solution, which was used as a lignin specific chromogenic agent. The pink part represents the xylem. The slide was photographed using an Olympus BX51 light microscope. (**A**) BcR; (**B**) BsR; (**C**) BmsR; (**D**) BmR; (**E**) BsmR.

After staining with lignin specific chromogenic agents, the xylem at the center of each fresh sample showed a distinct pink color. As shown in Figure 7, the xylem rays of *B. chinense* DC. and *B. scorzonerifolium* Willd. were denser, while the xylem rays of *B. smithii* Wolff and *B. marginatum* Wall. ex DC. were more scattered. Only the edges of the xylem of *B. marginatum* var. *stenophyllum* were neat, with a few rays visible inside the xylem, and many unknown dark green circular dot structures appeared in its phloem after staining.

**Figure 8:**
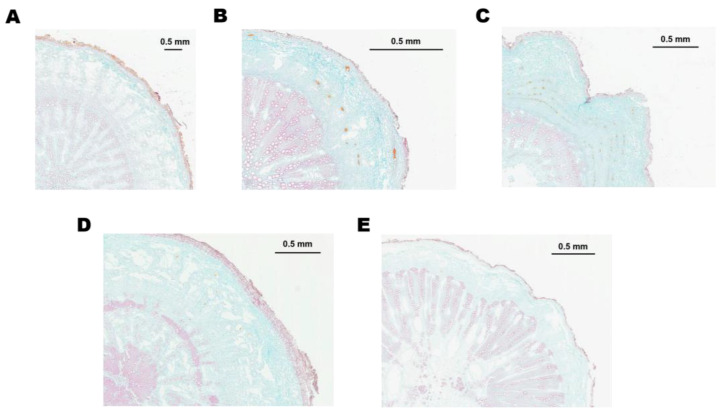
The microscopic structure of the root cross-sections of the five species/varieties. The samples were stained by 1% safranine and 0.5% fast green solution; the red part represents the nucleus and the lignified cell walls; the blue part represents the cytoplasm and the cellulose containing cell walls. The slides were photographed using an Olympus BX51 light microscope. (**A**) BcR; (**B**) BsR; (**C**) BmsR; (**D**) BmR; (**E**) BsmR.

The calculation of the area ratio of xylem to phloem showed that *B. chinense* DC. and *B. marginatum* Wall. ex DC. had the highest ratios of 0.3991 and 0.3643, respectively; *B. scorzonerifolium* Willd. was 0.3290, *B. smithii* Wolff was 0.2578, while *B. marginatum* var. *stenophyllum* had the lowest ratio of 0.2253, indicating that the proportion of phloem in *B. marginatum* var. *stenophyllum* is the highest. Previous studies have shown that the volatile oil and saikosaponins of the *Bupleurum* plants mainly come from their phloem. With safranine and fast green staining, we also found a large number of golden oil droplets in the phloem of *B. marginatum* var. *stenophyllum*, while hardly any were observed in *B. chinense* DC (Figure 8). Therefore, it is speculated that the high proportion of phloem might be the structural basis for the significant increase in volatile oil content in *B. marginatum* var. *stenophyllum* compared to other species.

## 3. Discussion

### 3.1. Substance Basis Difference between B. marginatum var. stenophyllum and Other Four Species

Modern pharmacological studies have shown that the volatile oil of *Bupleurum* has antipyretic, anti-inflammatory and other effects, and it is considered the pharmacodynamic material basis for Bupleuri Radix (“*Chaihu*” in Chinese) to mitigate fever [17]. *Chaihu injection* made from volatile oil of Bupleuri Radix is a commonly used medicine in the treatment of fever, which is listed in the national standard for Chinese patent drugs (NSCPD).

The sources of Bupleuri Radix (“*Chaihu*” in Chinese) listed in the *Pharmacopoeia of the People’s Republic of China* are *B. chinense* DC. and *B. scorzonerifolium* Willd. (State Pharmacopoeia Commission, 2020) and recognized as “*North Bupleurum*” and “*South Bupleurum*”. In addition, *B. marginatum* Wall. ex DC. and *B. smithii* Wolff are collected by multiple local standards. The dried roots of *B. marginatum* var. *stenophyllum* are neither included in Pharmacopoeia nor in local standards. At present, there is a phenomenon of misusing *B. marginatum* var. *stenophyllum* as Bupleuri Radix in the market. *B. marginatum* var. *stenophyllum* is a variant of *B. marginatum*. In the heatmap, there were obvious differences between *B. marginatum* var. *stenophyllum* and other samples in chemical composition. And our PCA results also showed that *B. marginatum* var. *stenophyllum* are different from the other six samples (Figure 3A,B).

In the Chinese tradition, *B. scorzonerifolium* Willd contains a high content of volatile oil, which gives it a strong odor and is called “*XiangChaihu*” [21]. Our results show that the volatile oil content of *B. scorzonerifolium* Willd and its wild species was the highest among all the samples, which was consistent with the results of Yu and Chen et al. [21,22]. In our results, the relative contents of lauraldehyde, *α*-pinene, *β*-pinene, and sabinene in BsR and BsR_W were significantly higher than other samples. Huo’s study suggested that lauraldehyde might be the effective components of the volatile oil of the *B. scorzonerifolium* Willd [23], and that sabinene, *β*-pinene, and *α*-pinene all had good anti-inflammatory activities [24,25,26]. In particular, the relative content of caproaldehyde in *B. chinense* DC. was higher than that in other samples. One of the effects of Bupleuri Radix recorded in the *Pharmacopoeia of the People’s Republic of China* is its mitigation of fever. Some studies have shown that the two chemical components, caproaldehyde and heptaldehyde, are closely related to the pharmacological effect of the volatile oil of Bupleuri Radix in mitigating fever. Some studies also use caproaldehyde and heptaldehyde as the basis for quality control of *Chaihu injection*, *Chaihu oral liquid*, and *Chaiqin soft capsules* [27,28,29]. This may be the reason why the antipyretic effect of the two species is better than that of *B. marginatum* Wall. ex DC. and *B. smithii* Wolff. The content of lauraldehyde in *B. smithii* Wolff was much lower than that in *B. scorzonerifolium* Willd, but the relative contents of *α*-pinene, *β*-pinene, and sabinene in *B. smithii* Wolff were significantly higher than in the other samples, which may be the reason why it was used for mitigating fever [30]. Although the volatile oil content of *B. marginatum* Wall. ex DC. was the lowest in all samples, its content of caproaldehyde and heptanaldehyde was relatively high, which may be the reason for its clinical use in mitigating fever [22].

Compared with other samples, there are some different metabolites with higher relative contents in *B. marginatum* var. *stenophyllum*. We found that dehydrodihydroionone is often used as a food additive (https://echa.europa.eu/) (accessed on 7 December 2023) and that 4′-methylvalerophenone is somewhat toxic. In addition, 1-phenylhexan-3-one and 3-hydroxyacetophenone are volatile compounds with high content in *B. marginatum* var. *stenophyllum*, which are somewhat toxic and may cause eye and skin irritation. If *B. marginatum* var. *stenophyllum*. is to be included in the medical standard in the future, it needs to be fully studied to prove its efficacy.

The chemical composition of plants in different growth processes and different states was different [2,31,32]. Traditionally, Bupleuri Radix is generally used in medicine after drying in the shade, so the chemical composition of the dried root can better explain the efficacy of the volatile oil of Bupleuri Radix. When the transcriptome and metabolome are analyzed together, usually, fresh roots are used in the transcriptome, and fresh or dried roots are used in the metabolome. We used the traditional drying method to dry the roots. In order to verify the reliability of the transcriptome data measured by fresh roots, we analyzed the volatile oils in fresh roots. Then, we compared the chemical composition of the fresh and dried roots. Our results showed that the relatively up-regulated compounds in the fresh roots and dry roots of *B. marginatum* var. *stenophyllum* did not change. This means that the differential genes we found are reliable (Figure S5 and Table S11).

Some studies have found that the contents of the same compound in different plants have different trends in the drying process [33,34]. We also found that, for example, after drying, the contents of *α*-pinene and *β*-pinene in BsR and BmsR increased, while the contents of these two compounds in BcR decreased; the content of heptanaldehyde in all species increased; and the content of lauraldehyde in BsR and BsmR increased, while the content in other species was similar or decreased. (Figure S5 and Table S11). That may be because some biological transformations take place during the natural drying of plants, such as oxidation, reduction, and acetylation reactions [35,36].

### 3.2. The Plant Basis Difference between B. marginatum var. stenophyllum and Other Four Species

This study presents, for the first time, the differences in volatile oil contents among medicinal species/varieties of the genus *Bupleurum*. *B. scorzonerifolium* Willd. showed the highest volatile oil content, followed by *B. smithii* Wolff and *B. marginatum* var. *stenophyllum*. *B. chinense* DC. and *B. marginatum* Wall. ex DC. showed the lowest volatile oil contents, which was less than 1/5 of *B. scorzonerifolium* Willd. We speculated that the reason for these significant differences is related to the geographical location of the plant habitat.

Volatile oil is also an important secondary metabolite product that both the above- and below-ground parts of the plant generate in response to external stimuli [37]. Recently, extensive studies have revealed its role in plant abiotic stress resistance, especially in cold resistance. It has been shown that volatile compounds could ensure the survival of plants under severe cold conditions by maintaining membrane integrity and fluidity through the accelerated synthesis of abundant unsaturated fat [38]. Studies in several medicinal plants, such as *Litsea cubeba*, *Camellia sinensis*, and *Astragalus mongholicus*, have shown that the levels of their volatile oil are most positively correlated with higher latitude or lower annual average temperature [37,39,40,41]. The evolution research of the genus *Juglans* through pan-genome analyses further revealed that in eastern black walnut, with the sharp reduction of the effective population during the glacial climate, there was a large number of InDel sites in the genes involved in fatty acid synthesis [42]. Together, all these pieces evidence confirmed that volatile components such as volatile oil are indispensable for the response of plants to cold.

In this study, the volatile oil contents exhibited significant positive correlation with latitude; that is, *B. scorzonerifolium* Willd., with the highest volatile oil content, is located in the region with the highest altitude and latitude (1800 m, 35°40′ N), *B. marginatum* Wall. ex DC., with the lowest volatile oil content, is located in the region with the low altitude and latitude (350 m, 29°45′ N), and so on (Table 2). The ranking of the annual average temperature of the above regions was also similar as that of altitude or content. Thus, the temperature difference based on altitude and latitude might be the important reason underlying the differences in volatile oil content among different *Bupleurum* species/varieties. Moreover, we demonstrated that for medicinal plants, in addition to the widely studied volatile oils in the above-ground parts, the volatile oils in the roots are also highly sensitive to environmental temperature.

Interestingly, in contrast to the ranking of volatile oil content, the odor analysis of dried samples using e-nose technology showed that the strongest response on the volatile organic compound sensor (S8 in Figure 1B) was from *B. marginatum* Wall. ex DC., which come from the region with the lowest latitude, while the weakest response was from *B. scorzonerifolium* Willd., which is located in the region with the highest latitude. This result is consistent with the fact that aromatic plants are more common in subtropical and tropical regions as they need to attract insect pollination over a longer reproductive period. The above results indicated that there are significant differences in the volatile compounds of the *Bupleurum* species/varieties, which might be related to the environmental temperature.

As shown by our metabolomic analysis (Figure 3D), there are sharp differences between *B. marginatum* var. *stenophyllum* and other species in the contents of many volatile oil components. To explore the underlying molecular mechanism, transcriptome sequencing was conducted, and numerous genes involved in the biosynthesis of volatile components were found to be only differentially expressed in *B. marginatum* var. *stenophyllum*, such as the members of *TPS*, *GPS*, and *DXS* families (Figure 6).

The biosynthesis pathway of terpenoids is usually divided into three stages: First, the C5 precursor (isopentenyl diphosphate, IPP) and its *cis*-*trans* isomer (dimeryl diphosphate, DMAPP) are generated through the MVA/MEP pathway, where HMGR and DXS are the rate limiting enzymes of the MVA and MEP pathways, respectively. The second stage is the generation of the core carbon skeletons of terpene, namely, direct precursors (FPP, GPP, GGPP, etc.). Finally, further modification of the carbon skeleton (oxidation-reduction, acylation, glycosylation, etc.) is carried out to ultimately generate different terpenoids. The first two stages are shared by all terpenoids; the third stage determines the structural diversity of terpenoids [2].

The volatile terpenoids are mainly monoterpenes and sesquiterpenes. GPS is responsible for catalyzing the synthesis of the monoterpenoid precursor GPP from IPP and DMAPP. TPS is located at the branching point of the isoprenoid biosynthesis pathway (IBP) and is a key enzyme in the synthesis of terpenoids. TPS can catalyze the formation of multiple products from a single substrate through subtle structural changes, which makes it a major contributor to the structural diversity of terpenoids in nature [43].

Based on transcriptome analysis and PCR analysis, it was found that the genes which are unique and widely present in *B. marginatum* var. *stenophyllum* (DXS, GPS, TPS) precisely cover the three stages of terpenoid synthesis. These results indicated that compared with other *Bupleurum* species, the involvement of a large number of new gene members at multiple key nodes of the monoterpene synthesis pathway in *B. marginatum* var. *stenophyllum* might be an important reason for its high volatile oil content and more complex composition. Comparative genomic studies of *Wurfbainia longiligularis* and *Wurfbainia villosa* revealed that there are significantly more TPS members involved in the biosynthesis of volatile monoterpene in the former species, which has relative lower medicinal value, and the tissue-specific expression patterns between the two species were also different [44]. Therefore, it is indicated that the excessive involvement of gene members at key nodes might lead to a shift in synthesis direction from the main synthesis pathway to the branching pathway, ultimately resulting in a more complex component composition.

## 4. Plant Materials and Methods

### 4.1. Plant Materials

The medicinal *Bupleurum* species/varieties that are mainly distributed in China were selected in this study, including *B. chinense* DC. (BcR), *B. scorzonerifolium* Willd. (BsR), *B. marginatum* var. *stenophyllum* (Wolff) Shan et Y. Li (BmsR), *B. marginatum* Wall. ex DC. (BmR), and *B. smithii* Wolff (BsmR), among which *B. chinense* DC. and *B. scorzonerifolium* Willd. are listed in the *Chinese Pharmacopoeia*, and *B. marginatum* Wall. ex DC. and *B. smithii* Wolff are included in different local standards in China. According to our review [1], all the sampling locations for the above species (BcR, BsR, BmR, BsmR)/varieties (BmsR is the varieties of BmR) are currently their optimal production areas. For both *B. chinense* DC. and *B. scorzonerifolium* Willd., their cultivated germplasm and the wild germplasm were collected from the same areas to avoid additional errors caused by geographical factors. The resources of the other three species/varieties were all cultivated germplasm. The detailed information of location, altitude, latitude, longitude, and growth years (all two years) were shown in Table 2, the soil type of all the sampling locations was sandy soil. All the plant materials were harvested in early October. After harvesting, the fresh roots were immediately frozen in liquid nitrogen as fresh samples; the remaining roots were dried in the shade to obtain the dried sample.

For the dried samples that were used for e-nose analysis, metabolomics analysis, and the hydrochloric acid phloroglucinol staining, the fully shaped, non-moldy, and disease-free dried roots were used. For *Bupleurum marginatum* var. *stenophyllum* (Wolff) Shan et Y. Li and the other four species/varieties (including the 2 planting types for *B. chinense* and *B. scorzonerifolium*, respectively), there were 7 groups in total. For each group, three dried roots were randomly selected and mixed as one sample, each group had three samples as three replicates; the biological replications for the dried samples that used for e-nose analysis was five.

For the fresh samples that were used for DNA bar-coding analysis, metabolomics analysis, transcriptome analysis, PCR analysis, and safranine and fast green staining, the fully shaped, healthy fresh roots were used. Same as the sampling way of dried samples, for each group, three dried roots were randomly selected and mixed as one sample; each group had three samples as three replicates.

### 4.2. DNA Barcoding Analysis

DNA bar-coding was used to identify whether the species/varieties of the plant material used are correct. One of the most suitable barcodes (ITS) of Bupleuri Radix was used for PCR amplification, and the primer sequences were shown in Table S1 [5]. DNA extraction of the dried roots, PCR amplification, sequencing, and sequence alignment were performed according to our previous studies [5]. The related ITS sequences that are used as controls were obtained from GenBank (*B. chinense* DC.: MH703316.1; *B. scorzonerifolium* Willd.: MK258717.1; *B. marginatum* var. *stenophyllum*: MK258708.1; *B. marginatum* Wall. ex DC.: MK258709.1; *B. smithii* Wolff: MZ029691.1). A phylogenetic tree was constructed using the neighbor-joining algorithm (NJ tree) with 1000 bootstrap replicates.

### 4.3. E-Nose Detection of the Dried Roots

For each sample, a total of 0.5 g dried roots (unground, not powder) was sealed in an e-nose sample bottle for 24 h, followed by heating the sample in a water bath at 60 °C for 30 min and then cooling it down at room temperature for 10 min. The syringe was used to filter out the excess water vapor of the bottle and the aspirated 20 mL gas from the bottle, which was then injected into an e-nose detector (Thinksenso, Hangzhou, China) for automatic sampling and determination. The sample detection parameters were as follows: the carrier gas was clean, dry, and three-level filtered air; the test flow rate was 0.6 L/min; the duration of a single cleaning was 60 s; and the duration of a single testing was 60 s. The detection pattern was cross testing.

### 4.4. HS–SPME–GC–MS Detection of Volatiles

Extraction was carried out via automatic headspace solid-phase microextraction (HS-SPME, SPME arrow, CTC Analytics AG, Zwingen, Switzerland) for gas chromatography coupled with mass spectrometry (GC-MS, Model 8890-7000D; Agilent, Santa Clara, CA, USA) analysis. Liquid nitrogen was added into each dried or fresh sample. A 0.5 g sample was accurately weighed and placed in headspace sampling bottles. A quality control (QC) sample was prepared by mixing all dried or fresh samples, and three QC samples were inserted before, during, and after the samples. Methyl 2-hydroxybenzoate-3,4,5,6-d4 (C/D/N ISOTopes, Montreal, QC, Canada) was used as an internal standard. Saturated NaCl solution and 10 µL (50 µg/mL) of the internal standard solution were added, respectively. The vials were sealed using crimp-top caps with TFE-silicone headspace septa. At the time of SPME analysis, each vial was placed at 60 °C for 5 min, then a 120 µm DVB/CWR/PDMS fiber (Agilent, USA) was exposed to the headspace of the sample for 15 min at 60 °C, and then the fiber head was inserted into the GC-MS inlet and desorbed at 250 °C for 5 min.

The capillary column was DB-5MS (30 m × 0.25 mm × 0.25 μm, Agilent, USA). The inlet temperature was 250 °C, and the non-shunt injection mode was adopted. High-purity helium (≥99.999%) was used as carrier gas to flow at the speed of 1.2 mL/min, and the solvent delay was 3.5 min. In the heating stage, the initial temperature (40 °C) was kept for 3.5 min and increased to 100 °C at a rate of 10 °C/min in 6 min; then, it ws raised to 180 °C at a rate of 7 °C/min and raised to 280 °C a rate of 25 °C/min (held for 5 min). The energy was 70 eV in the electron impact mode (EI). The quadrupole mass detector, ion source, and transfer line temperatures were set at 150, 230, and 280 °C, respectively. The ion mode selected ion monitor (SIM) was scanning mode.

The original data are processed by MassHunter software (B.08.00, Agilent). The volatile compounds were identified by comparing the mass spectra with the local database (MWGC, Wuhan Metware Biotechnology Co., Ltd., Wuhan, China). In short, one quantitative ion and two-to-three qualitative ions were selected for each compound. If the retention time of the detected peak is consistent with the reference and all the selected ions appear after background subtraction, then the compound is determined [45]. The relative content of individual volatile components was calculated from the peak area, and the peak area was standardized according to the internal standard (IS) (Area_standardized_ = Area_sample_/Area_IS_).

### 4.5. Statistical Analysis

R project (www.r.project.org, accessed on 30 January 2024) was used for visualization. Principal component analysis (PCA) and hierarchical clustering analysis (HCA) were performed to analyze metabolites. The significance of the difference in metabolites among five species/varieties was evaluated via orthogonal projections to latent structure-discriminant analysis (OPLS-DA) and volcano plots.

### 4.6. Transcriptome Analysis

Three biological repeats of the fresh roots of five species/varieties were used for transcriptome sequencing. RNA extraction, cDNA library construction, and Illumina sequencing (Illumina HiSeq 2100 platform, Tokyo, Japan) were performed according to Wang et al. [46]. The clean reads were obtained by removing adapter sequences and low-quality reads, and they were de novo assembled using Trinity with the default parameters. BUSCO software (V3) was used to test and evaluate the accuracy and integrity of the assembled results files [2]. Gene function was annotation based on SwissProt, Nt, KO, GO, Pfam, and KOG databases with an E-value of 10~(−5), respectively. Gene expression levels were calculated via FPKM. The gene with an adjusted *p*-value < 0.05 and |log_2_FoldChange| > 1 was assigned as differentially expressed. The cluster Profiler R package (v3.16) was used to perform GO and KEGG enrichment analysis. The *B. chinense* DC. group (BcR) was set as the control for either DEG analysis or KEGG analysis.

### 4.7. Gene Expression Analysis

The expression levels of the representative DEGs were quantified using qRT-PCR with the TransStart Top Green qPCR Super Mix (Aidlab, Beijing, China) and Light Cycler 96 system (Roche, Basel, Switzerland). The primers were listed in Table S1. Three biological replicates with three technical replicates were conducted for each sample. The reference gene was Actin. The 2^−ΔΔCt^ method was used for calculating relative expression levels.

### 4.8. Observation of the Microstructure of the Root Cross-Section

For the observation of xylem and phloem, the fresh roots were sliced by hand; then, we added 2~3 drops of hydrochloric acid phloroglucinol solution (lignin-specific chromogenic agent) to the root cross-section. After dyeing for 2 min, the excess dye was washed off with distilled water, and the slices were placed on the glass slide then photographed with an Olympus BX 51 optical microscope. The images were exported to the Digimizer software (6.0), and the ratio of xylem to root area was calculated by using this software.

Safranine and fast Ggeen staining was conducted to observe the oil droplets. The 3 mm fresh roots were placed in the embedding mold, subjected to 70%, 80%, 90%, 95%, and 100% ethanol gradient dehydration and 65% wax immersion. After the wax block cooled down, it was sliced into 4 μm sections using the Ultra-Thin Semiautomatic Microtome (Leica, Vizna, Germany). The wax slides were then spread and flattened on a glass slide then placed at 65 °C for 1 h to dry the moisture. Afterwards, 1% saffron and 0.5% solid green staining were performed sequentially. After neutral balata fixation, the observation-of-results process was the same as that of hydrochloric acid phloroglucinol staining.

## 5. Conclusions

In this study, we found that compared with other legitimate *Bupleurum* species/varieties, *B. marginatum* var. *stenophyllum* showed a relatively high level of volatile oil content and the greatest differences with others in terms of both the composition and the contents of hydrocarbons, esters, and ketones. The involvement of a large number of new gene members at multiple key nodes of the monoterpene synthesis pathway (DXS, GPS, TPS) in *B. marginatum* var. *stenophyllum* might have contributed to its high volatile oil content and more complex composition. The high proportion of phloem might underline the structural basis for its abundant oil droplets in phloem. The huge differences in the environmental temperature of the plant habitat are speculated to be the main reasons for the overall differences among these five species/varieties based on the analysis of e-nose and metabolome sequencing. Taken together, this study once again confirmed the importance of plant origin in the development of alternative resources to the genus *Bupleurum*, as well as other traditional Chinese medicines, and provided new insights into the research strategy for volatile oil components in numerous rhizome medicinal plants.

## Figures and Tables

**Table 1:** The top 20 characteristic differential metabolites.

No.	Retention Time (min)	Compounds	Molecular Formula	Type
1	9.37	2-Methyltetrahydrothiophen-3-one	C_5_H_8_OS	Sulfur compounds
2	17.23	Dehydrodihydroionone	C_13_H_20_O	Ketones
3	18.72	(*Z*)-*γ*-bisabolene	C_15_H_24_	Hydrocarbons
4	18.71	Geranyl isobutyrate	C_14_H_24_O_2_	Esters
5	18.73	Cubebanol	C_15_H_26_O	Alcohols
6	18.75	(-)-*α*-Alaskene	C_15_H_24_	Hydrocarbons
7	19.27	N-Benzyloxy-2-carbomethoxyaziridine	C_11_H_13_NO_3_	Nitrogen compounds
8	19.27	4-Hydroxyphenylacetic acid	C_8_H_8_O_3_	Acids
9	16.90	Cedrene	C_15_H_24_	Hydrocarbons
10	17.22	4-Hydroxybenzyl alcohol	C_7_H_8_O_2_	Alcohols
11	18.72	4′-Methoxypropiophenone	C_10_H_12_O_2_	Ketones
12	15.02	(2-Nitroethyl)benzene	C_8_H_9_NO_2_	Nitrogen compounds
13	19.27	4-Aminobenzoic acid	C_7_H_7_NO_2_	Acids
14	19.27	1-(4-Methoxyphenyl)-3-methyl-1-pentanone	C_13_H_18_O_2_	Ketones
15	17.23	Geosmin	C_12_H_22_O	Alcohols
16	16.90	2-methylene-4,8,8-trimethyl-4-vinyl-bicyclo[5.2.0]nonane	C_15_H_24_	Hydrocarbons
17	16.90	*α*-Gurjunene	C_15_H_24_	Hydrocarbons
18	17.96	Acoradiene	C_15_H_24_	Hydrocarbons
19	19.27	8,14-Cedranoxide	C_15_H_24_O	Ethers
20	17.23	Myosmine	C_9_H_10_N_2_	Nitrogen compounds

**Table 2:** The sampling information of all the plant materials.

Species	Abbreviation	Type	Producing Area	Latitude and Longitude	Altitude
*B. chinense* DC.	BcR	Cultivated	Chencang District, Baoji City, Shanxi Province, China	107°37′ E 34°35′ N	700 m
	BcR_W	Wild	Chencang District, Baoji City, Shanxi Province, China	107°37′ E 34°35′ N	700 m
*B. scorzonerifolium* Willd.	BsR	Cultivated	Lindian County, Daqing City, Heilongjiang Province, China	124°87′ E 47°17′ N	150 m
	BsR_W	Wild	Lindian County, Daqing City, Heilongjiang Province, China	124°87′ E 47°17′ N	150 m
*B. marginatum* var. *stenophyllum* (Wolff) Shan et Y. Li	BmsR	Cultivated	Lintao County, Dingxi City, Gansu Province, China	103°86′ E 35°40′ N	1800 m
*B. marginatum* Wall. ex DC.	BmR	Cultivated	Rong County, Zigong City, Sichuan Province, China	104°42′ E 29°45′ N	350 m
*B. smithii* Wolff	BsmR	Cultivated	Pinshun County, Changzhi City, Shanxi Province, China	113°44′ E 36°20′ N	1200 m

## Data Availability

The data presented in this study are available in article and Supplementary Materials.

## Supplementary Materials

The following supporting information can be downloaded at https://www.mdpi.com/article/10.3390/molecules29112561/s1: Figure S1: The phylogenetic relationships of the 5 species/varieties of Bupleuri Radix; Figure S2: Overview of metabolites of fresh samples; Figure S3: Volcano plots of differential metabolites in fresh samples; Figure S4: The qRT-PCR analysis of part of the differentially expressed genes; Figure S5: Volcano plots of differential metabolites of dried and fresh samples; Table S1: Primer information; Table S2: Data of E-Nose; Table S3: List of the compounds in dried samples; Table S4: Differential metabolites between BmsR and other dried samples; Table S5: List of the compounds in fresh samples; Table S6: Differential metabolites between BmsR and other fresh samples; Table S7: Summary of sequencing quality; Table S8: Unigene sequence homology search against the public databases; Table S9: Summary of transcript and unigene assembly data; Table S10: List of the annotations of the DEGs; Table S11: Differential metabolites between dried and fresh samples of BsmR.
