# Supplementary material for: Comprehensive Analysis Reveals the Difference in Volatile Oil between Bupleurum marginatum var. stenophyllum (Wolff) Shan et Y. Li and the Other Four Medicinal Bupleurum Species
Source: Molecules. 2024 May 29;29(11):2561. doi: 10.3390/molecules29112561 (PMC11173446; doi:10.3390/molecules29112561)
Supplement: Supplementary file 1 [file molecules-29-02561-s001.zip › Figure S1-S5.pdf]

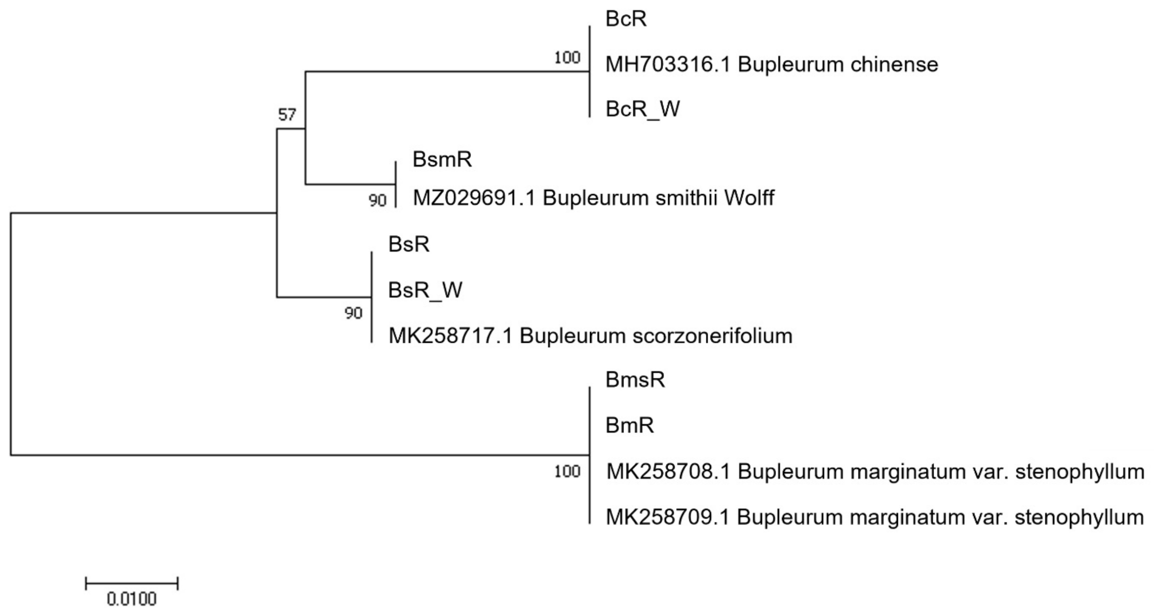

Figure S1 The phylogenetic relationships of the 5 species/varieties of Bupleuri Radix. The bootstrap support values are given on the branches.

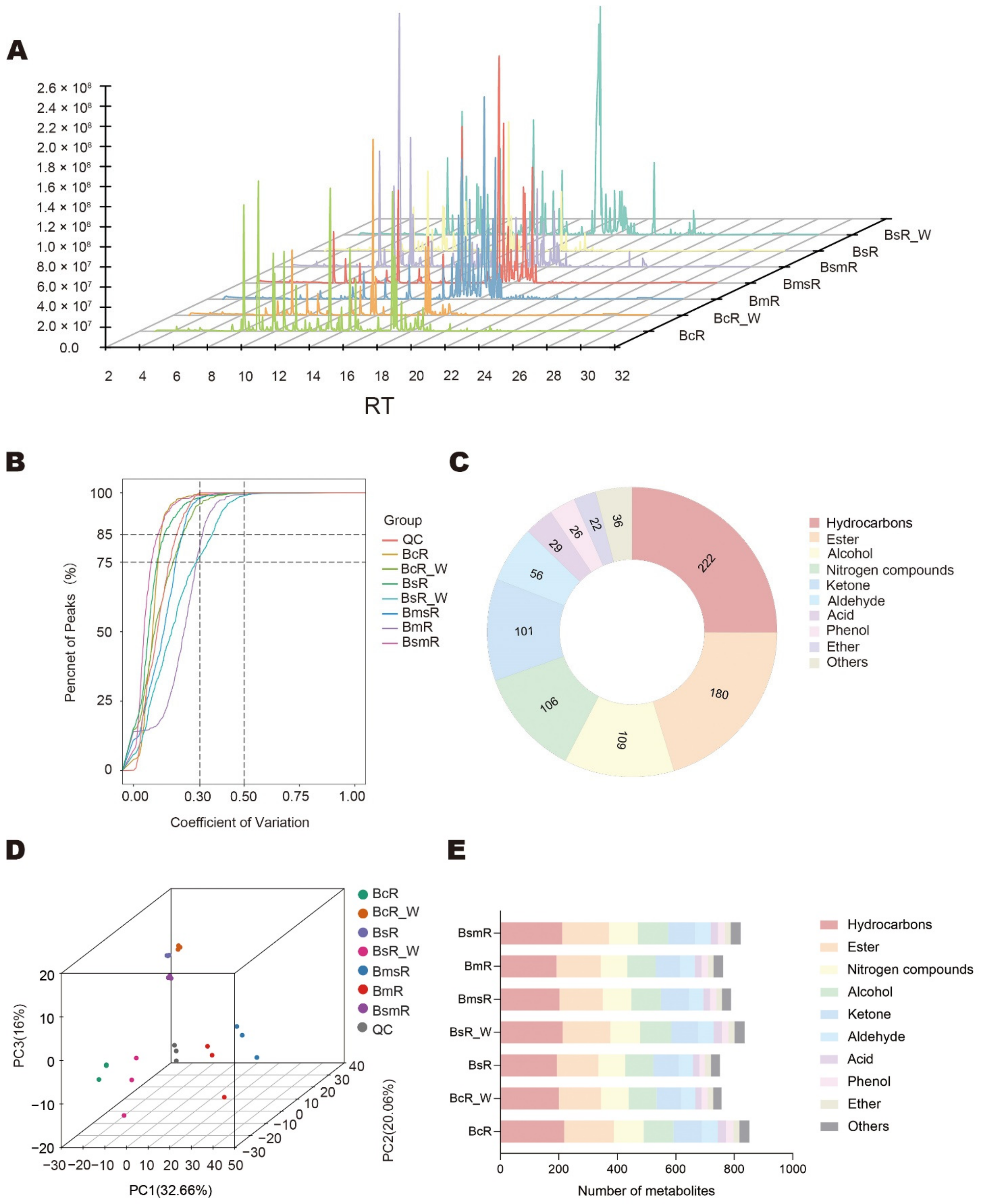

Figure S2 Overview of metabolites of fresh samples. Total ion chromatogram (TIC) of fresh samples (A) and quality control (QC) sample with the coefficient of variation (CV) of fresh samples (B), less

than 0.3 accounted for more than 75% of the samples, indicating that the experimental data were very stable. (C) Doughnut chart of the number of different volatile metabolites types of fresh samples. (D) The 3D PCA plot of fresh samples. (E) Distribution of different types of metabolites in fresh samples.

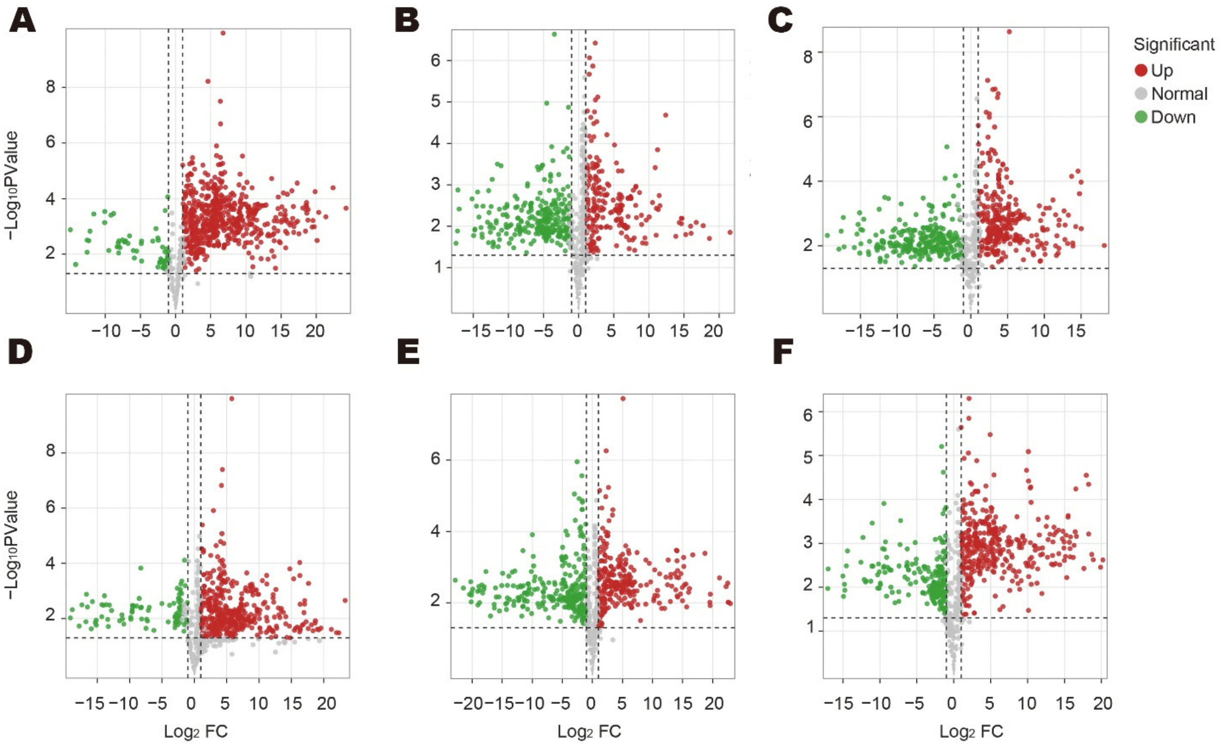

Figure S3 Volcano plots of differential metabolites in fresh samples. BcR vs BmsR (A); BcR_W vs BmsR (B); BsR vs BmsR (C); BsR_W vs BmsR (D); BmR vs BmsR (E); BsmR vs BmsR (F).

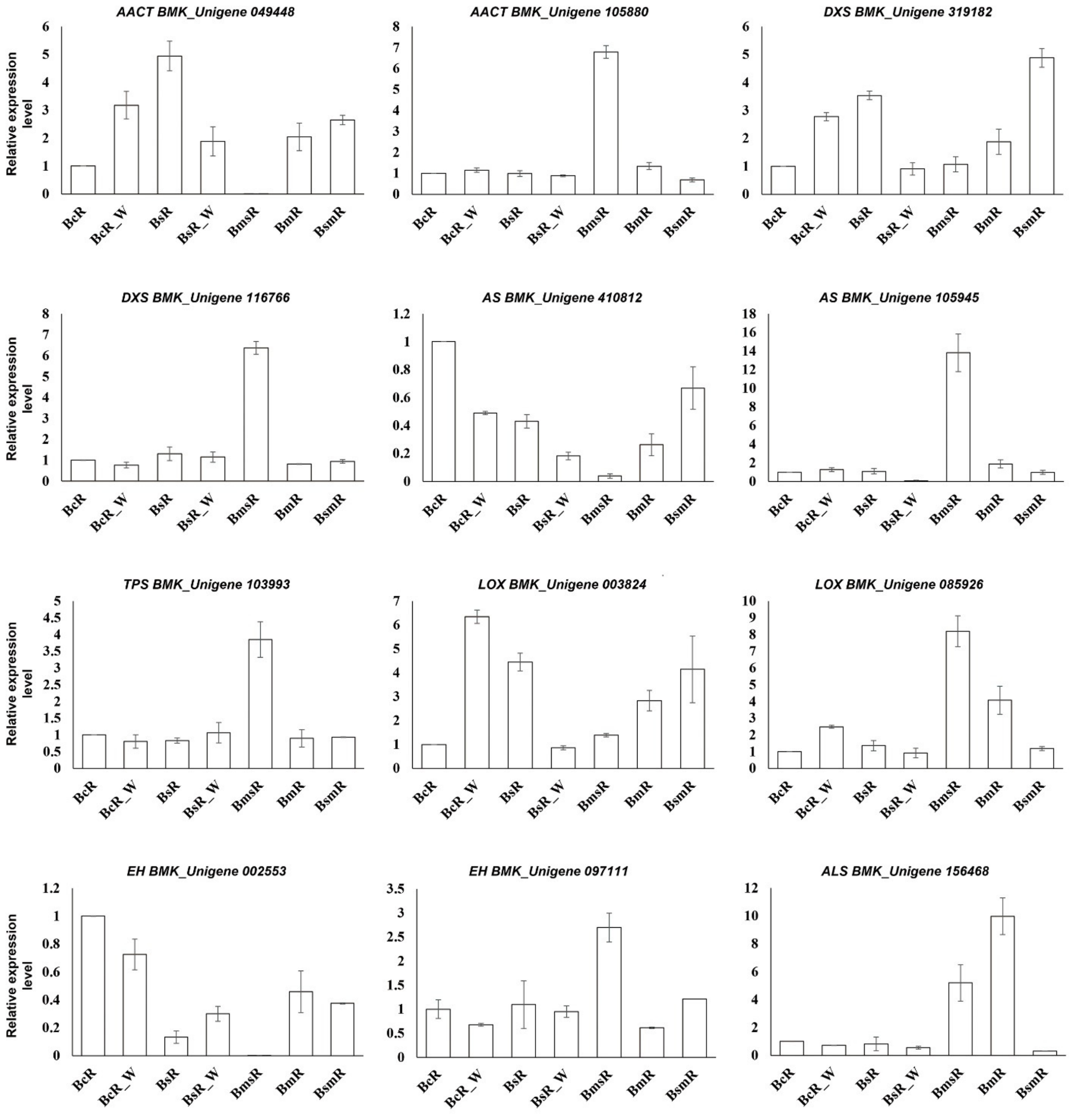

Figure S4 The qRT-PCR analysis of part of the differentially expressed genes. The fresh samples of *B. chinense* was used as control.

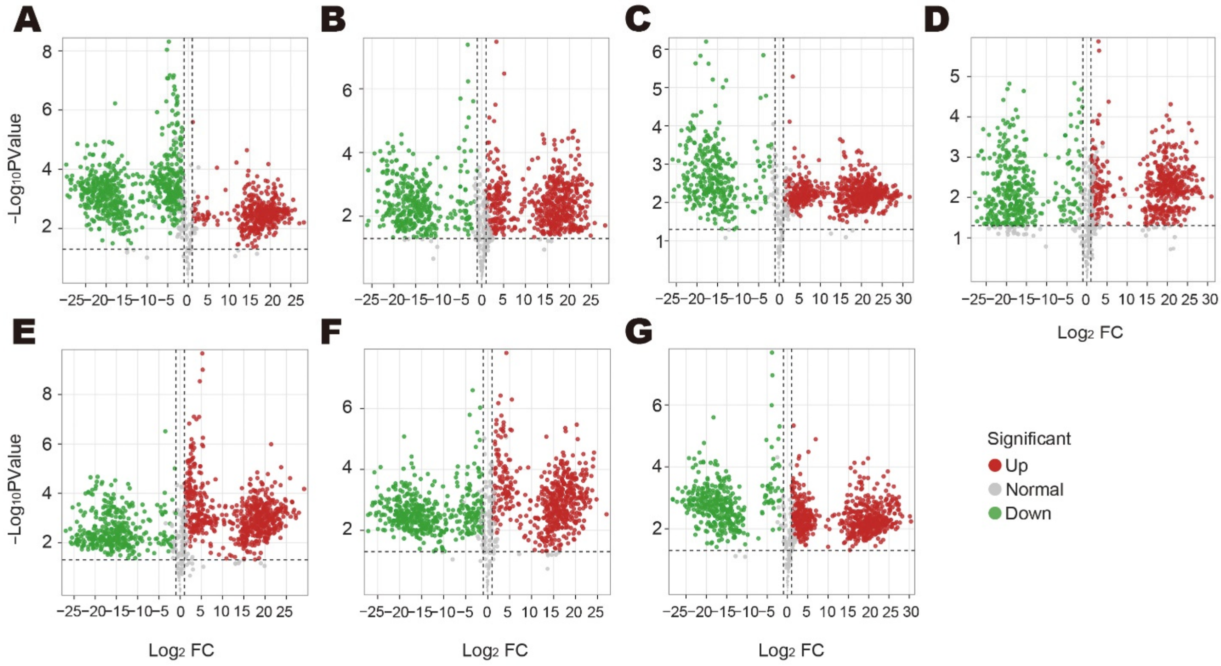

Figure S5 Volcano plots of differential metabolites of dried and fresh samples. BcR(A); BcR_W (B); BsR (C); BsR_W (D); BmsR (E); BmR (F); BsmR (G).
