# Supplementary material for: Comprehensive Analysis Reveals the Difference in Volatile Oil between Bupleurum marginatum var. stenophyllum (Wolff) Shan et Y. Li and the Other Four Medicinal Bupleurum Species
Source: Molecules. 2024 May 29;29(11):2561. doi: 10.3390/molecules29112561 (PMC11173446; doi:10.3390/molecules29112561)
Supplement: Supplementary file 1 [file molecules-29-02561-s001.zip › Tables S1.pdf]

Table S1 Primer information

Gene ID	Forward primer sequence (5'>3’)	Reverse primer sequence (5'>3’)
ITS2	ATGCGATACTTGGTGTGAAT	GACGCTTCTCCAGACTACAAT
18S	CAACCATAAACGATGCCGA	AGCCTTGCGACCATACTCC
BMK_Unigene_319182, EH	AGGTGGTATTCTCGGGTA	AAACTGGAACGGGATCAA
BMK_Unigene_003824, LOX	TAGCCTGTTGGTCTTGATTGC	GTTAGCATCCGCCTTCTTCAA
BMK_Unigene_049448, AACT	GCAGTGGAGCAAGACTTT	TAGAACGCCCAACCATTG
BMK_Unigene_085926, LOX-2	CGGATGAGGAGTACATTGG	CAGAACTAGGTGTGAGTAGC
BMK_Unigene_097111, EH-2	GCCAACCGATACACCAGAT	ATTCACCTTGTCAGGAAATTGC
BMK_Unigene_103993, TPS	GATTTGGCATCTGGGAATT	CTGAAGCAAGTGAAGAAGG
BMK_Unigene_105880, AACT-2	GGACATCCTCTAGGTTGC	CCGATCAGTCTGGTAAAGAA
BMK_Unigene_105945, AS	CGCCATTGGTAAGGTGTCT	TGGAAGTTGCCATCACTCT
BMK_Unigene_116766, DXS	TTGCTTGTGCTGATCCTTAC	GTGCCACCAAACTGTTCA
BMK_Unigene_156468, ALS	TTGGACAACAACAGATGTG	GCCTTATAGAACCGATCCT
BMK_Unigene_319182, DXS	GCAAATGGGATGTGATGG	TTATCTCCGCAAGCAACT
BMK_Unigene_410812, AS	TTGTGCCATTTGGAGGAG	CAATGAGGCGAAGTGGAA
