# Supplementary material for: Comprehensive Analysis Reveals the Difference in Volatile Oil between Bupleurum marginatum var. stenophyllum (Wolff) Shan et Y. Li and the Other Four Medicinal Bupleurum Species
Source: Molecules. 2024 May 29;29(11):2561. doi: 10.3390/molecules29112561 (PMC11173446; doi:10.3390/molecules29112561)
Supplement: Supplementary file 1 [file molecules-29-02561-s001.zip › Tables S7.pdf]

Table S7 Summary of sequencing quality.

Sample	Clean reads	Clean bases	GC content	%⩾Q30
R-1-1	20515631	6.14G	43.21%	94.73%
R-1-2	19163005	5.74G	43.13%	93.95%
R-2-1	21169721	6.34G	43.12%	94.12%
R-2-2	20486227	6.13G	42.88%	94.76%
R-3-2	21517007	6.44G	42.85%	94.02%
R-3-3	21994912	6.59G	43.27%	94.11%
R-4-1	24051289	7.20G	42.86%	94.77%
R-4-2	23580455	7.06G	42.82%	94,48%
R-4-3	19906393	5.96G	42.83%	94.60%
R-5-1	25491886	7.63G	43.18%	94.26%
R-5-2	20721416	6.21G	43.05%	94.52%
R-5-3	22463116	6.73G	42.92%	94.24%
R-6-2	24815652	7.43G	42.55%	92.09%
R-6-3	20257635	6.06G	42.67%	93.83%
R-7-1	19594714	5.87G	43.09%	94.48%
R-7-3	19054510	5.71G	43.16%	94.88%
