# Supplementary material for: Comprehensive Analysis Reveals the Difference in Volatile Oil between Bupleurum marginatum var. stenophyllum (Wolff) Shan et Y. Li and the Other Four Medicinal Bupleurum Species
Source: Molecules. 2024 May 29;29(11):2561. doi: 10.3390/molecules29112561 (PMC11173446; doi:10.3390/molecules29112561)
Supplement: Supplementary file 1 [file molecules-29-02561-s001.zip › Tables S8.pdf]

Table S8 Unigene sequence homology search against the public databases.

	200-300 bp	300-500 bp	500 bp-1kb	1-2kb	>2kb
Unigenes	29912	15269	12761	21959	20059
Proportion	29.91%	15.28%	12.77%	21.97%	20.07%
