# Supplementary material for: Comprehensive Analysis Reveals the Difference in Volatile Oil between Bupleurum marginatum var. stenophyllum (Wolff) Shan et Y. Li and the Other Four Medicinal Bupleurum Species
Source: Molecules. 2024 May 29;29(11):2561. doi: 10.3390/molecules29112561 (PMC11173446; doi:10.3390/molecules29112561)
Supplement: Supplementary file 1 [file molecules-29-02561-s001.zip › Tables S9.pdf]

Table S9 Summary of transcript and unigene assembly data.

	Annotated Number	Length≤300	Length≥1000
Annotated in COG	21863	3805	16199
Annotated in GO	56228	13145	35536
Annotated in KEGG	46059	9507	31183
Annotated in KOG	38866	8218	26006
Annotated in Pfam	52190	11103	36300
Annotated in Swissprot	45450	9449	31930
Annotated in TrEMBL	65470	16368	40704
Annotated in eggNOG	52819	11817	35962
Annotated in NR	71495	18640	41100
Annotated in all Databases	73380	19338	41251
